# Paralumbar Spine Disease as a Cause of Low Back Pain in Older Adults

**DOI:** 10.7759/cureus.53983

**Published:** 2024-02-10

**Authors:** Fumiaki Fujihara, Kyongsong Kim, Toyohiko Isu, Juntaro Matsumoto, Koichi Miki, Masanori Isobe, Tooru Inoue, Hiroshi Abe

**Affiliations:** 1 Department of Neurosurgery, Hakujyuji Hospital, Fukuoka, JPN; 2 Department of Neurological Surgery, Chiba Hokusoh Hospital, Chiba, JPN; 3 Department of Neurosurgery, Kushiro Rosai Hospital, Kushiro, JPN; 4 Department of Neurosurgery, Faculty of Medicine, Fukuoka University, Fukuoka, JPN

**Keywords:** middle cluneal nerve, superior cluneal nerve, paralumbar spine disease, older adults, low back pain

## Abstract

Introduction

Low back pain (LBP) is a major contributor to decreases in the ability to perform activities of daily living (ADL) in older adults. Paralumbar spine disease (PLSD) is a common cause of LBP. We aimed to investigate the causes of LBP, including PLSD, among older adults.

Methods

Among 744 consecutive patients with LBP, 75 patients (10.1%) aged >80 years (25 males and 50 females) were included. The average patient age was 83.9 years. All patients were evaluated using lumbar magnetic resonance imaging (MRI) and radiography to diagnose the causes of LBP. PLSD was diagnosed based on clinical symptoms, palpation, and the effects of the block.

Results

Eleven patients (11/75, 14.7%) had acute osteoporotic vertebral fractures. Twenty-eight of the remaining 64 patients exhibited decreased LBP with oral medication, and six (6/75, 8.0%) exhibited lumbar spinal canal stenosis on MRI. PLSD was suspected in 19 of the remaining 30 cases based on clinical symptoms and palpation. Blocks were effective in 16 patients with PLSD, which involved superior cluneal nerve entrapment (SCN-E) in eight patients (10.7%), middle cluneal nerve entrapment (MCN-E) in nine patients (12.0%), sacroiliac joint (SIJ) pain in five patients (6.7%), and gluteus medius muscle (GMeM) pain in three patients (4.0%). The average numerical rating scale (NRS) scores for pain changed from 7.5 ± 1.5 before treatment to 1.3 ± 0.9 at discharge (p < 0.05).

Conclusion

Osteoporotic acute vertebral fracture (14.7%) was identified as the cause of LBP in older adults. Block therapy for PLSD may aid in the diagnosis and treatment of non-specific LBP.

## Introduction

Low back pain (LBP) is a common finding in older adults [1], the prevalence of which ranges from 21% to 75% [2] and increases until the age of 80 years, following which a slight decrease is observed [3]. Several studies have reported that LBP leads to a high incidence of functional limitations and decreased ability to perform activities of daily living (ADL) in older adults [4,5]. In 2005, the United States spent more than $100 billion on LBP-related healthcare, and two-thirds of these costs were indirect due to lost wages and reduced productivity [6]. Historically, research on LBP has primarily focused on young and adult populations, whereas little attention has been given to the older adult population. However, age is a well-known risk factor for chronic LBP [7], and age-related changes in the body may perpetuate LBP in older adults, increasing associated medical expenses in this population.

In older adults, LBP commonly results from age-related physical changes in lumbar spinal structures (spondylolisthesis, spinal canal stenosis, osteoporotic vertebral fracture, etc.) and the muscles and ligaments surrounding the lumbar spine. These age-related physical changes may be associated with differences in the incidence of diseases leading to LBP between younger and older patients.

Previous studies have reported that the cause of LBP is unknown in approximately 85% of patients, in which case the condition is classified as “non-specific LBP” [8,9]. Non-specific LBP cannot be clearly diagnosed or treated based on radiological findings. Paralumbar spine disease (PLSD), which includes cases of superior/middle cluneal nerve entrapment (SCN-E/MCN-E), sacroiliac joint (SIJ) pain, and gluteus medius muscle (GMeM) pain, is classified as non-specific LBP. PLSD is a term used to refer to diseases that cause LBP around the lumbar spine, buttocks, and leg [10]. Radiological images cannot be used to diagnose PLSD, which is instead identified based on tenderness and the effectiveness of blocks. Given that some patients may develop PLSD as a manifestation of failed back surgery syndrome after lumbar spine surgery [11], minimally invasive strategies such as blocks may be particularly beneficial for the diagnosis and treatment of LBP in older adults.

Some authors have reported that the incidence of PLSD is relatively common, with SCN-E and MCN-E occurring in 1.6%-14% [12-14] and 13.1% [14,15] of LBP patients, respectively. Such studies have also indicated that PLSD is more common among older adults [13-15] and patients with vertebral fractures [13,16]. Thus, the incidence of PLSD as a cause of LBP may be higher in older patients. Clarifying this incidence may aid in the treatment of LBP in the older adult population. Therefore, in the present study, we aimed to investigate the incidence of LBP due to PLSD in older adults treated at our institution.

## Materials and methods

Patients

This study was approved by the ethics committee of our hospital. Written informed consent for inclusion in this study was obtained from all patients.

Between January 2016 and January 2019, 744 consecutive patients consulted our institution for LBP (383 of the 744 patients were included in our earlier studies [14,15] that focused on diagnosing PLSD in LBP patients). Among them, 75 patients (10.1%) aged >80 years (25 males and 50 females) were included in this study. The patients with severe dementia, malignant tumors, and iliac crest harvesting for grafting were excluded. The average patient age was 83.9 years (range: 80-93 years). All patients were evaluated using lumbar magnetic resonance imaging (MRI) and radiography at the outpatient clinic, and the patients with severe lumbar diseases (pyogenic spondylitis, acute vertebral fracture, etc.) were urgently admitted. The patients without such diseases were first treated on an outpatient basis with oral medications such as nonsteroidal anti-inflammatory drugs, pregabalin, and tramadol.

Diagnostic criteria for PLSD

Based on clinical symptoms, including palpation findings and LBP, we performed block therapy for the diagnosis and treatment of patients with suspected PLSD. SCN-E, which occurs due to the entrapment of the SCN near the iliac crest, was diagnosed based on worsening pain with movement and Tinel-like signs observed at the entrapment points. The diagnoses of SCN-E were made when direct local anesthetic blockage was observed at the site of the Tinel-like sign, resulting in a pain reduction of at least 75% [16]. MCN-E refers to LBP involving the MCN area, which worsens with movement. In such cases, the entrapment point is located 35 mm caudal to the posterior superior iliac spine and slightly lateral to the iliac crest edge. The diagnoses of MCN-E were made when local MCN block resulted in a pain reduction of more than 50% [17-19]. SIJ pain refers to pain occurring around the lumbogluteal area. The diagnoses of SIJ pain were made when SIJ injection resulted in a pain reduction of at least 75% [20]. The diagnoses of GMeM pain were made when the nerve block resulted in pain reduction at the trigger point of the GMeM [21].

Evaluation methods

We evaluated the severity of LBP based on the numerical rating scale (NRS) scores, Japanese Orthopaedic Association (JOA) scores, and Roland-Morris Disability Questionnaire (RDQ) results at the time of admission and discharge. The self-reported NRS scores for LBP severity ranged from 0 (no pain) to 10 (severe pain). Improvements in symptoms were judged to have occurred when NRS scores had improved by 3 or more points [22]. JOA scores range from 0 to 29, with a score of 29 indicating an absence of symptoms. Scores are determined based on the physician ratings of subjective symptoms, objective findings, the effect of LBP on the patient’s ADL function, and bladder and bowel dysfunction. The RDQ reflects the patient’s subjective assessment of the impact of LBP on ADL function. RDQ scores range from 0 to 24, with higher scores indicating more severe pain.

Statistical analysis

All continuous variables (age and length of hospitalization) were expressed as the mean with the range (minimal to maximal), while discrete variables (NRS, JOA, and RDQ scores) were expressed as the mean with the standard deviation (SD). GraphPad Prism version 8.0 (GraphPad Software, San Diego, CA) was used for subsequent statistical analyses. NRS, JOA, and RDQ scores were analyzed using two-tailed Wilcoxon matched-pairs signed-rank tests. The significance level was set at p < 0.05.

## Results

Relationship between vertebral fracture and LBP

**Figure 1:**
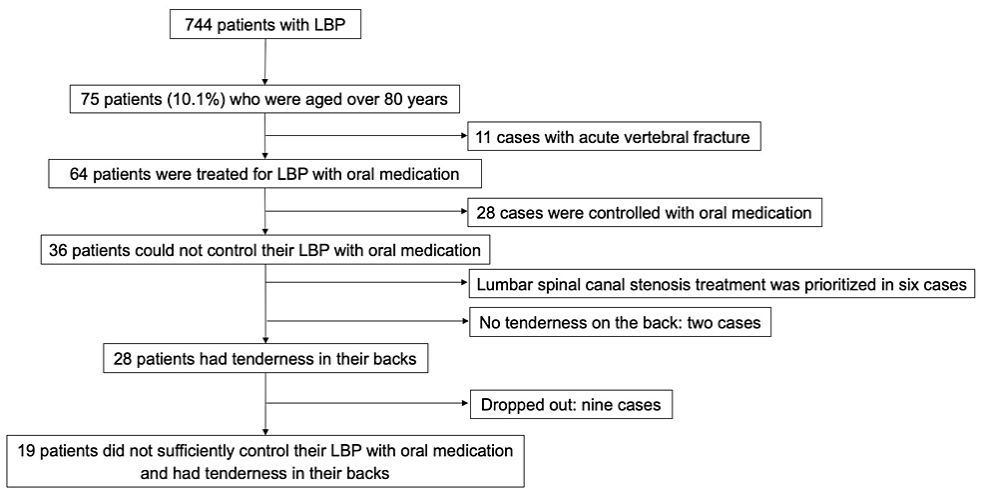
Flow chart for diagnosing low back pain (LBP)

There were no cases of vertebral infection, metastasis of malignant tumors, or infection in our study. The cause of LBP was acute osteoporotic vertebral fracture in 11 of the 75 patients (11/75, 14.7%) (Figure 1). The average age of the patients with osteoporotic acute vertebral fracture (five males and six females) was 85.9 years (range: 81-93 years). Excluding those with osteoporotic acute vertebral fracture, chronic osteoporotic vertebral compression fracture was observed in 22 patients (22/64, 34.3%) with radiography studies at the outpatient clinic. Among the 11 patients with osteoporotic acute vertebral fracture, chronic osteoporotic vertebral compression fracture was observed in six cases (6/11, 54.5%).

In the remaining 64 patients without acute vertebral fractures, LBP was treated with oral medication, which decreased symptoms in 28 cases (Figure 1). Six of these patients (6/28, 21.4%) had chronic osteoporotic compression fractures. Among the other 36 patients in whom LBP could not be controlled with oral medication, chronic osteoporotic compression fracture was observed in 16 cases (16/36, 44.4%).

Among the 36 patients with uncontrolled LBP despite treatment with oral medication, six had difficulty performing ADLs due to lower limb pain and intermittent claudication caused by lumbar spinal canal stenosis as observed on lumbar MRI. The treatment for these patients was prioritized. Two patients underwent posterior decompression at our institution, while the remaining four requested treatment at other hospitals.

Of the remaining 30 patients, two had no tenderness in their lower back or buttocks. The other 28 patients reported tenderness in the lower back, suggesting PLSD as a cause of LBP. Nine patients requested consecutive treatment at local hospitals near their homes and dropped out of the study due to difficulty remaining at our hospital given the distance or their poor physical condition. The other 19 patients were admitted for PLSD evaluation due to the effects of intractable LBP on ADL function (Figure 1).

Evaluation of PLSD in 19 patients

PLSD evaluations were performed on 19 patients (13 males and six females) with an average age of 83.9 years (range: 80-89 years). During hospitalization, one patient required treatment for transient delirium, but this did not affect LBP treatment.

SCN block attenuated LBP pain in eight patients (8/75, 10.7%), two of whom were diagnosed with SCN-E alone, while the other six patients were diagnosed with coexisting PLSDs (Table 1). MCN block was effective in nine patients (9/75, 12.0%). Three of these nine patients were diagnosed with MCN-E alone, while the other six patients were diagnosed with coexisting diseases. SIJ block was effective in five patients (5/75, 6.7%), two of whom were diagnosed with SIJ pain alone, while the other three patients were diagnosed with coexisting diseases. GMeM block was effective in three patients (3/75, 4.0%), all of whom were diagnosed with coexisting diseases.

**Table 1:** Incidence of paralumbar spine disease in older adults with low back pain SCN-E, superior cluneal nerve entrapment; MCN-E, middle cluneal nerve entrapment; SIJ, sacroiliac joint; GMeM, gluteus medius muscle

	n	(%)
SCN-E	8/75	10.7%
Alone	2/8	
Coexisting disease		
MCN-E	3/8	
SIJ pain	1/8	
GMeM pain	2/8	
MCN-E	9/75	12.0%
Alone	3/9	
Coexisting disease		
SCN-E	3/9	
SIJ pain	2/9	
GMeM pain	1/9	
SIJ pain	5/75	6.7%
Alone	2/5	
Coexisting disease		
SCN-E	1/5	
MCN-E	1/5	
GMeM pain	2/5	
GMeM pain	3/75	4.0%
Alone	0/3	
Coexisting disease		
SCN-E	2/3	
MCN-E	1/3	

**Figure 2:**
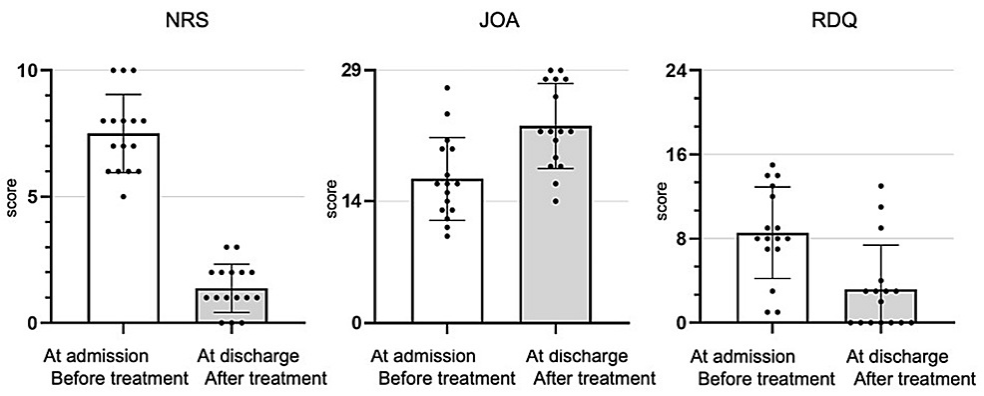
Outcome of block treatment for paralumbar spine disease in 19 patients Outcome assessment: to evaluate the clinical outcomes of block treatment for paralumbar spine disease in 19 low back pain patients using NRS, JOA, and RDQ, we compared NRS, JOA, and RDQ scores between admission and discharge. The boxes and error bars represent the mean value and standard deviation, respectively; individual data points are also plotted. A significant difference was observed in all evaluations. The mean values with p < 0.05 are considered statistically significant NRS, numerical rating scale; JOA, Japanese Orthopedic Association; RDQ, Roland-Morris Disability Questionnaire

Based on the results of block treatment for PLSD, 16 (84.2%, 16/19) patients were diagnosed with PLSD as the cause of LBP. Among them, nine patients were diagnosed with more than one PLSD. The average number of block treatments required during hospitalization was 4.9 ± 1.5 (range: 3-8). Pain scores at the time of admission and discharge (average hospital stay, 15.9 ± 9.8 days; range, 8-48 days) were compared to assess the effectiveness of treatment (Figure 2). The average NRS score changed from 7.5 ± 1.5 before treatment to 1.3 ± 0.9 at discharge (p < 0.05). The average JOA scores increased from 16.6 ± 5.0 before treatment to 22.7 ± 5.4 at discharge (p < 0.05), while the average RDQ scores decreased from 8.6 ± 4.6 before treatment to 3.2 ± 4.5 at discharge (p < 0.05).

## Discussion

Specific LBP

In our study, acute osteoporotic vertebral fractures (14.7%) were identified as the cause of LBP in 11 older adults, an incidence higher than that reported among all patients with LBP in previous studies (3%-4%) [8,23]. Lumbar spinal stenosis represented the cause of LBP in six cases (8%), and there were no cases of lumbar disc herniation, vertebral infection, or metastasis in the current study. The incidence of specific LBP was 22.7% in older adults included in our study, which is similar to that indicated in previous reports (15.0%-21.2%) [8,9,23].

Previous studies have reported an association between chronic osteoporotic compression fractures and LBP [24,25]. Osteoporotic compression fracture has also been highlighted as a risk factor for LBP, and the limitation of activity increases with the number of vertebral fractures [25]. Vertebral deformity originating from osteoporosis leads to poor posture, with an increase in thoracic kyphosis, straightening of lumbar lordosis, and S-curve lumbar and thoracic scoliosis. Poor posture can therefore cause LBP originating from the ligaments, muscles, and fascia that support the spine and from the intervertebral joints [26].

In this study, chronic osteoporotic compression fracture was common among patients in whom LBP could not be controlled with oral medication (44.4%), while it was less common among those in whom pain control was achieved via oral treatment (21.4%). Our analysis also suggested an association between osteoporosis and intractable LBP, indicating that preventing osteoporotic compression fractures can attenuate the progression of LBP and ADL limitations in patients with osteoporosis.

Incidence of PLSD in older adults

PLSDs such as SCN-E, MCN-E, SIJ pain, and GMeM pain cannot be diagnosed based on radiological findings but are instead diagnosed based on clinical symptoms, palpation results, and the effects of block therapy. Therefore, they are classified as non-specific LBP. Suzuki et al. [23] reported non-specific LBP in 23.1% (74/320) of cases, including fascial lumbago (56/320, 17.5%) and SIJ pain (18/320, 5.6%). The incidence of PLSDs such as SCN-E and MCN-E tends to increase with age [13,15,17,27,28]. In addition, postural abnormalities in Parkinson’s disease or vertebral body fracture are reported to result in increasing muscle tonus in the thoracolumbar fascia and cause the contraction of the latissimus dorsi and gluteus maximus, which are associated with the incidence of superior cluneal nerve entrapment [29,30]. However, the incidence of SIJ pain has not been reported to correlate with age [31]. In this study, 21.3% of all patients (16/75) exhibited improvements in LBP following PLSD treatment. These results suggest that it is important to consider PLSD when treating LBP in older adults.

Treatment for PLSD

Block therapy for PLSD is minimally invasive and can contribute to both pain reduction and diagnosis [12,32]. In previous studies, SCN blocks were successful in 34%-100% of the patients with SCN-E [13,17,29,33], while MCN blocks were successful in 44% of the patients with MCN-E [15]. Such studies have reported that the patients with SCN-E required two or three blocks in 47% and 25% of cases, respectively [13], while those with MCN-E required a median of two blocks [15]. It is important to note that it is not uncommon for a single block therapy to result in the incomplete control of symptoms. Even when single treatments are effective for analgesia, it should be assumed that the disease will continue to affect the patient, and a second or subsequent treatment should be considered. In our series, the average number of block therapies in the 16 patients with PLSD was 4.9. Additionally, PLSD is often associated with other diseases [15]. In the present study, nine patients had more than one PLSD, and comprehensive treatment contributed to symptomatic improvement.

PLSDs may not relieve pain with block therapy alone. The surgical treatment of SCN-E, MCN-E, and GMeM pain can be performed less invasively under local anesthesia, which is useful for older adult patients [34]. But older age and diabetes mellitus have been reported as risk factors for postoperative complications [35], and caution is required.

Limitations

Our study had some limitations. First, the incidence of PLSD may have been higher than reported because only the patients who did not improve following treatment with oral medication were evaluated for PLSD. However, this may be less important, as these patients responded to medication and did not require PLSD treatment. Second, we focused on short-term treatment results for PLSD. If sufficient LBP relief is not available after several block treatments, surgery is considered for the next treatment. Third, it has been suggested that changes in alignment due to vertebral body fractures may induce LBP. However, regarding the alignment change before and after the onset of LBP, it is difficult to survey whether the change in alignment is related to LBP because there are few cases in which X-rays have been taken before and after the onset of LBP. This is an issue for the future.

## Conclusions

In our study, osteoporotic acute vertebral fracture (14.7%) was identified as the cause of LBP in 11 older adults, and chronic osteoporotic compression fracture was common among patients in whom LBP could not be controlled with oral medication. These results suggest an association between osteoporosis and intractable LBP. Given that over 20% of the patients exhibited improvements in LBP following block therapy for PLSD, this strategy may aid in the diagnosis and treatment of non-specific LBP. Importantly, it is not uncommon for a single block therapy to result in incomplete symptom control, and PLSD is often associated with other diseases. Thus, comprehensive strategies are required when diagnosing and treating PLSD.
